# Sodium oxybate with reduced-dose propofol for anesthesia induction in elective spine surgery: a randomized trial

**DOI:** 10.3389/fmed.2026.1970835

**Published:** 2026-10-13

**Authors:** Jianfeng Yu, Cuiping Yu, Nanling Wu, Kaixuan Zhao, Yimeng Xia, Ting Fan

**Affiliations:** 1 Department of Anesthesiology, Tsinghua University Yuquan Hospital (Tsinghua University Hospital of Integrated Traditional Chinese and Western Medicine), Beijing, China; 2 Department of Anesthesiology, Ruijin Hospital, Shanghai Jiao Tong University School of Medicine, Shanghai, China

**Keywords:** anesthesia induction, bispectral index, gamma-hydroxybutyrate, hypotension, propofol, randomized controlled trial, sodium oxybate, spine surgery

## Abstract

**Background:**

Sodium oxybate has an established sedative action, but its contribution to contemporary propofol-based anesthesia remains incompletely characterized. We evaluated induction hemodynamics, propofol use, bispectral index (BIS), and early recovery in elective spine surgery.

**Methods:**

In this single-center randomized trial, 127 patients received propofol alone (P; *n* = 62) or propofol plus sodium oxybate (PS; *n* = 65). Planned induction doses were propofol 2.2 versus 1.2 mg/kg, with oxybate 30 mg/kg in PS; recorded propofol doses were lower in both groups. The primary outcome was the mean arterial pressure (MAP) trajectory across 13 induction landmarks. Maximum-likelihood mixed models were supplemented by unstructured-covariance and baseline-adjusted analyses.

**Results:**

The primary group-by-time interaction was not significant (*P* = 0.607; unstructured covariance, *P* = 0.719). The baseline-adjusted average post-baseline MAP difference was 1.7 mmHg (PS minus P; 95% confidence interval [CI], −2.0–5.5). Systolic-pressure-defined hypotension occurred in 37/62 versus 32/65 patients (risk ratio, 0.82; 95% CI, 0.60–1.14; risk difference, −10.4 percentage points; 95% CI, −26.8–6.7). Recorded total propofol use was 31.4% lower (751.3 versus 515.4 mg; difference, −235.9 mg; 95% CI, −366.0 to −105.9). Maintenance use was also lower under unblinded BIS-guided titration. Post-intubation BIS <40 occurred in 2/62 versus 22/65 patients. The mean surgery-end-to-extubation interval was 10.5 versus 13.6 min (difference, 3.1 min; 95% CI, 0.9–5.2), although both medians were 10 min. Vasoactive-treatment documentation was incomplete.

**Conclusions:**

The oxybate-containing strategy was associated with substantially lower propofol use, lower BIS values, and a longer mean extubation interval; hemodynamic superiority was not demonstrated. These findings define the observed effects and trade-offs of the combined regimen under BIS-guided care and inform further evaluation of dose selection, hypnosis monitoring, and recovery.

**Clinical Trial Registration:**

https://www.chictr.org.cn Chinese Clinical Trial Registry, ChiCTR2400080686.

## 1 Introduction

Preventing hypotension remains a central challenge during propofol induction. Propofol reduces vascular resistance and stressed venous volume (1), and intraoperative hypotension is associated with postoperative organ injury (2). Spine surgery adds hemodynamic demands related to positioning and procedure duration (3). Alternative induction and maintenance regimens have therefore been evaluated in cervical and prone spine surgery (4, 5). Whether reducing propofol exposure improves blood-pressure control depends on the accompanying drugs and clinical management.

Sodium oxybate, the sodium salt of gamma-hydroxybutyrate (GHB), has sedative effects involving GABA-B and GHB-related mechanisms (6). Earlier studies evaluated GHB during spinal anesthesia (7) and linked its clinical sedative effects to BIS changes in children (8). Its contribution to contemporary propofol-based general anesthesia remains less well defined, particularly when clinicians use BIS to guide dosing. We conducted a randomized trial in elective spine surgery to test whether adding sodium oxybate to lower-dose propofol improved the induction MAP trajectory. Secondary outcomes characterized drug exposure, BIS, and early recovery within the same treatment strategy.

## 2 Materials and methods

### 2.1 Study design and participants

This prospective, single-center, parallel-group randomized trial was conducted at Tsinghua University Yuquan Hospital, Beijing, China. The ethics committee approved the study (2024KY039), and written informed consent was obtained from participants or their legally authorized representatives before enrollment. The study followed the principles of the Declaration of Helsinki and Good Clinical Practice guidelines. The trial was prospectively registered with the Chinese Clinical Trial Registry (ChiCTR2400080686) on 5 February 2024, before enrollment of the first participant. Reporting was guided by CONSORT 2025 (9, 10).

The stated eligibility criteria were age 40–80 years and elective spine surgery under general anesthesia with planned postoperative patient-controlled intravenous analgesia. Exclusions included preoperative communication difficulties, spinal deformity surgery, severe hepatic impairment, renal failure requiring renal replacement therapy, and participation in another trial. Severe bradycardia, sick sinus syndrome, and second-degree or higher atrioventricular block without a pacemaker were also exclusions. Seven randomized patients fell outside the stated age range: five in P and two in PS. Six were aged 36–38 years and one was aged 82 years. All remained in the intention-to-treat population; an age-restricted sensitivity analysis included 120 patients meeting the age criterion.

### 2.2 Randomization and masking

Approximately 30 min before anesthesia, assignment was determined by a computer-generated number: odd numbers indicated P and even numbers indicated PS. A coordinator uninvolved in anesthetic management or outcome assessment prepared the trial drugs. Access to the allocation sequence before assignment was not documented, leaving allocation concealment uncertain. Attending anesthesiologists were unblinded; postoperative assessors were unaware of allocation.

### 2.3 Interventions and perioperative management

Planned induction regimens were intravenous propofol 2.2 mg/kg in P and propofol 1.2 mg/kg plus sodium oxybate 30 mg/kg in PS. The oxybate injection contained 2.5 g in 10 mL. Analyses used recorded administered amounts, distinguished from planned doses. The dosing-weight convention, administration order and rate, and reasons for dose deviations were unavailable. The highest recorded oxybate dose was 2.5 g; a prespecified dose cap was not documented.

Induction co-medications included sufentanil and rocuronium, but patient-level doses were unavailable. Maintenance used propofol, sufentanil or remifentanil, and rocuronium as clinically indicated, with a BIS target of 40–60. The reported protocol specified norepinephrine titration whenever blood pressure fell by more than 20% from baseline. The trigger variable and adherence to this rule were not verifiable. Free-text notes documented several vasoactive agents; these were audited by patient, agent, and landmark. The notes lacked the completeness, concentrations, and durations required to reconstruct cumulative exposure.

Routine monitoring included electrocardiography, blood pressure, pulse oximetry, nasopharyngeal temperature, end-tidal carbon dioxide, BIS, pulse pressure variation (PPV), and urine output. Blood pressure was measured noninvasively or invasively as clinically indicated. Raw EEG, burst suppression ratio, signal quality index, and electromyographic activity were unavailable. Postoperative patient-controlled analgesia comprised hydromorphone and tropisetron diluted to 100 mL, with a 2-mL/h background infusion, 2-mL bolus, and 15-min lockout. Drug concentrations were unavailable.

### 2.4 Outcomes and recorded landmarks

The primary outcome was the MAP trajectory across 13 induction landmarks. These comprised baseline, study-drug administration, five nominal 1-min post-administration readings, intubation, and five nominal 1-min post-intubation readings. Baseline was a single preinduction reading. Hypotension was defined as any systolic blood pressure (SBP) < 80 mmHg or a ≥ 30% SBP decrease from baseline. Supportive outcomes included nadir MAP, absolute and percentage MAP changes, any MAP <65, <60, or <55 mmHg, and any MAP decrease ≥20%. We also counted MAP readings <65 mmHg. These summaries retained the original 13-landmark window, including baseline. Peak post-intubation MAP was the maximum from intubation through 5 min thereafter. The intubation MAP response was this peak minus MAP at 5 min after study-drug administration.

An exploratory area below MAP 65 mmHg used actual recorded clock times and piecewise-linear interpolation, with explicit integration of threshold crossings. Patients with duplicate-minute or reversed timestamps were excluded from this calculation alone. Landmarks were not treated as equally spaced minutes. The integral summarizes the sampled pressure trajectory and cannot capture unmeasured episodes.

Secondary outcomes included propofol exposure, remifentanil, fluids, potassium, BIS, heart rate, and postoperative outcomes. Weight normalization used recorded body weight. Maintenance propofol was also divided by body weight and total anesthesia duration, yielding exposure per anesthesia-hour rather than an actual maintenance infusion rate. BIS <40 was assessed separately during 1–5 min after intubation and at four maintenance landmarks: incision, wound closing, skin suturing, and surgery end. Mean maintenance BIS, within-patient SD, and the proportion of these four readings outside 40–60 were calculated. BIS was treated as a processed EEG index, without assuming equivalent hypnotic depth across regimens.

Full-course analyses used 19 common landmarks: the 13 induction readings, incision, wound closing, skin suturing, surgery end, extubation, and recorded exit. An ambiguously labeled turning landmark was excluded because its clinical meaning was inconsistent or unverified. Turning prone was not separately recorded, and the exit label did not distinguish operating-room exit from PACU discharge. The surgery-end-to-extubation interval was calculated from recorded timestamps and analyzed as an exploratory recovery-related measure.

Numeric rating scale (NRS) pain scores were recorded on postoperative days 1–3; rest-versus-movement conditions were unspecified. Delirium and postoperative nausea and vomiting (PONV) were recorded as binary outcomes, without verifiable instruments, observation windows, or severity assessments. Structured postoperative awareness assessment was unavailable. Potassium was analyzed using observed baseline and extubation values and complete paired changes. Systematic sampling 15–30 min after dosing was unavailable.

### 2.5 Statistical analysis and data audit

Analyses retained all 127 patients in their assigned groups, using available observations for each outcome. Continuous outcomes are summarized as mean ± SD, with mean differences (PS minus P) and 95% confidence intervals (CIs). Skewed outcomes also use median [interquartile range]. Welch tests compare means; Mann–Whitney tests provide distributional comparisons for threshold counts, reconstructed area, and extubation interval. Binary outcomes use two-sided Fisher exact tests, Wilson intervals for proportions, Newcombe intervals for risk differences, and log-scale risk-ratio intervals when both groups have events. Baseline characteristics are descriptive. Secondary, pointwise, and nonprespecified analyses are exploratory, without multiplicity adjustment.

The primary random-intercept model used maximum likelihood (ML), with group, categorical time, and their interaction. A 12-degree-of-freedom likelihood-ratio test compared models with and without interaction. Sensitivity analyses used a marginal repeated-measures model with an unstructured 13 × 13 covariance matrix. Constrained longitudinal data analysis (cLDA) imposed a common baseline mean and estimated the equally weighted average of 12 post-baseline group contrasts. Its Wald CIs were model-based. Gaussian generalized estimating equations (GEE), using working independence and patient-cluster robust covariance, assessed data-quality sensitivities and full-course trajectories. Supplementary Table S2 specifies the models and estimands.

All 1,651 induction MAP, heart-rate, and BIS entries were populated. Across 19 common landmarks, one surgery-end MAP value was missing, leaving 2,412 MAP readings and 2,413 heart-rate and BIS readings. Outcome-specific denominators are reported; no values were imputed. Source MAP values were retained in the primary analysis. Sensitivity analyses masked values outside their paired SBP–diastolic BP range, using zero and 5-mmHg tolerances. These rules identify internal inconsistencies without determining which component is erroneous. Supplementary Table S5 documents the source-data issues and their analytical handling.

Enrollment of approximately 120 patients was planned pragmatically, without a formal *a priori* sample-size calculation or noninferiority margin. No prospectively specified MAP difference, variance assumptions, or power target was documented. Effect estimates and CIs guide interpretation of precision. Unstructured-covariance, cLDA, GEE, data-quality, clock-based area, and extubation-interval analyses were not prespecified and are exploratory. Analyses used Python, NumPy, SciPy, and statsmodels; technical details are provided in the Supplementary Material.

## 3 Results

### 3.1 Participants and administered doses

All 127 randomized patients received their assigned intervention and completed follow-up: 62 in P and 65 in PS (Figure 1). Individual outcome fields contained missing values. Baseline characteristics are shown in Table 1; MAP averaged 100.8 ± 19.0 versus 105.3 ± 18.9 mmHg. Anterior cervical surgery accounted for 14/62 and 15/65 patients, respectively.

**Figure 1:**
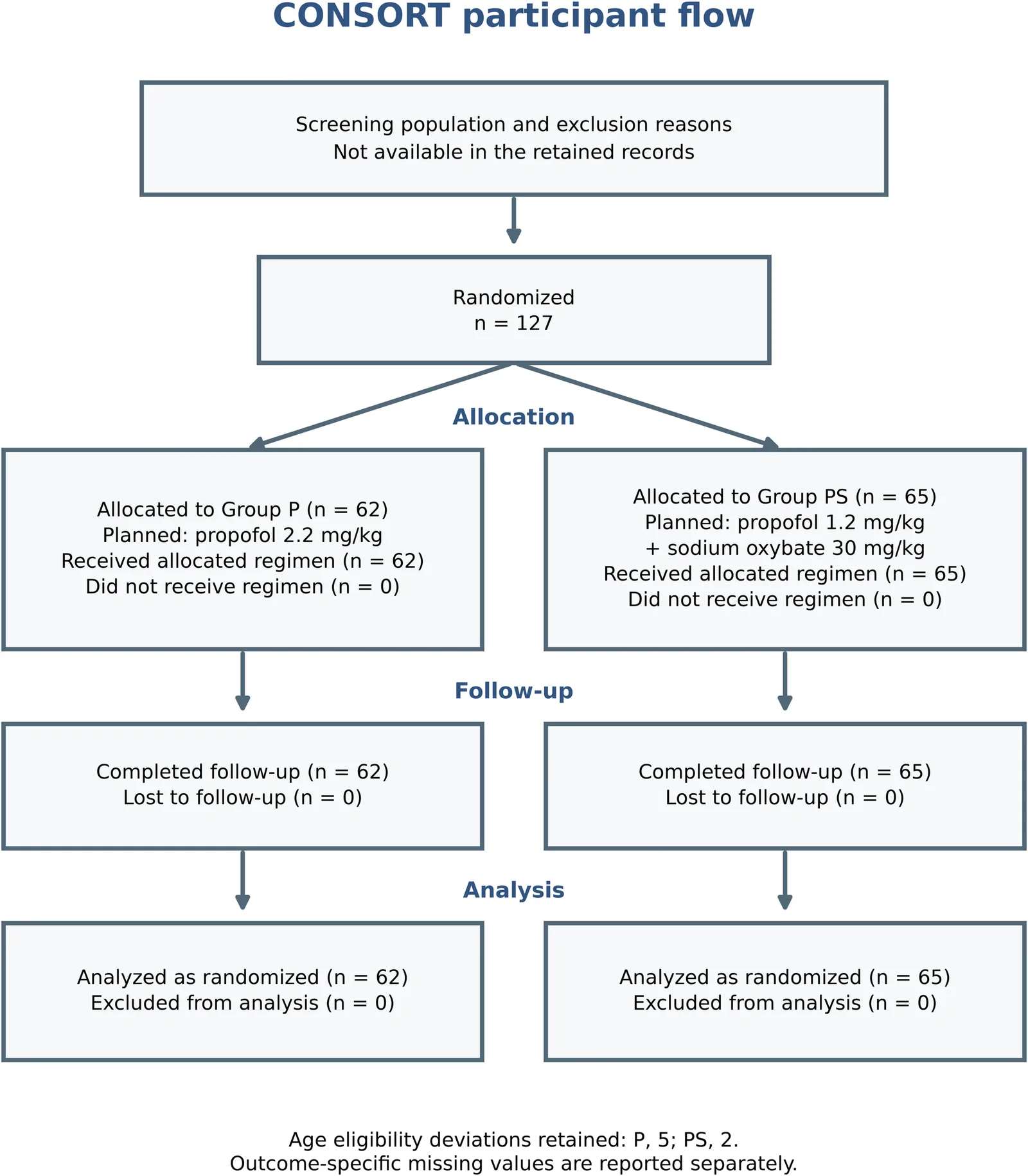
Participant flow. The screening population and reasons for exclusion before randomization were not available. All 127 randomized patients were retained in the intention-to-treat analysis. Completion of follow-up does not imply complete individual outcome fields. Seven patients outside the stated age range remained in the main analysis.

**Table 1:** Baseline characteristics.

Characteristic	Group P (*n* = 62)	Group PS (*n* = 65)
Age (years)	58.4 ± 11.1 (*n* = 62)	60.1 ± 8.7 (*n* = 65)
Height (cm)	166.8 ± 10.8 (*n* = 62)	167.3 ± 8.9 (*n* = 65)
Weight (kg)	72.8 ± 15.7 (*n* = 62)	70.2 ± 11.0 (*n* = 65)
Male sex	41/62 (66.1%)	44/65 (67.7%)
ASA class 1	17/62 (27.4%)	19/65 (29.2%)
ASA class 2	43/62 (69.4%)	44/65 (67.7%)
ASA class 3	2/62 (3.2%)	2/65 (3.1%)
Anterior cervical surgery	14/62 (22.6%)	15/65 (23.1%)
Posterior cervical surgery	18/62 (29.0%)	20/65 (30.8%)
Lumbar surgery	30/62 (48.4%)	30/65 (46.2%)
Baseline MAP (mmHg)	100.8 ± 19.0 (*n* = 62)	105.3 ± 18.9 (*n* = 65)
Baseline heart rate (beats/min)	72.6 ± 13.3 (*n* = 62)	72.4 ± 11.0 (*n* = 65)
Baseline BIS	94.9 ± 3.5 (*n* = 62)	95.1 ± 3.3 (*n* = 65)
Baseline PPV (%)	5.2 ± 1.7 (*n* = 62)	5.4 ± 1.9 (*n* = 65)
Baseline potassium (mmol/L)	3.71 ± 0.40 (*n* = 61)	3.59 ± 0.25 (*n* = 65)

Values are mean ± SD with observed n, or counts/denominators (%). No baseline significance tests were performed. ASA, American Society of Anesthesiologists; BIS, bispectral index; MAP, mean arterial pressure; PPV, pulse pressure variation.

Recorded induction propofol averaged 130.8 ± 32.6 mg in P (*n* = 61) and 70.4 ± 11.3 mg in PS (*n* = 65). Patient-level weight-normalized doses were 1.82 ± 0.29 and 1.00 ± 0.05 mg/kg, below the planned 2.2 and 1.2 mg/kg (Table 2). One induction-dose entry in P was missing. Sodium oxybate averaged 2.06 ± 0.31 g (range, 1.5–2.5 g), equivalent to 29.40 ± 2.04 mg/kg (range, 19.48–32.05). All five PS patients weighing >83.33 kg received 2.5 g, corresponding to <30 mg/kg. The rationale for this dosing pattern was undocumented.

**Table 2:** Recorded duration and anesthetic exposure.

Outcome	Group P	Group PS	MD (95% CI)	*P* value
Recorded anesthesia duration (min)	120.8 ± 43.1 (*n* = 62)	122.5 ± 59.5 (*n* = 65)	1.7 (−16.5 to 19.9)	0.853
Recorded surgery duration (min)	88.6 ± 36.4 (*n* = 62)	91.8 ± 56.1 (*n* = 65)	3.2 (−13.3 to 19.8)	0.701
Induction propofol (mg)	130.8 ± 32.6 (*n* = 61)	70.4 ± 11.3 (*n* = 65)	−60.5 (−69.2 to −51.7)	<0.001
Induction propofol (mg/kg)	1.82 ± 0.29 (*n* = 61)	1.00 ± 0.05 (*n* = 65)	−0.82 (−0.89 to −0.74)	<0.001
Maintenance propofol (mg)	618.5 ± 401.9 (*n* = 62)	445.0 ± 319.0 (*n* = 65)	−173.5 (−301.5 to −45.6)	0.008
Recorded total propofol (mg)	751.3 ± 412.2 (*n* = 62)	515.4 ± 319.3 (*n* = 65)	−235.9 (−366.0 to −105.9)	<0.001
Total propofol (mg/kg)	10.32 ± 4.83 (*n* = 62)	7.50 ± 5.07 (*n* = 65)	−2.83 (−4.57 to −1.09)	0.002
Maintenance propofol per anesthesia-hour (mg/kg/h)	4.21 ± 1.76 (*n* = 62)	3.01 ± 0.92 (*n* = 65)	−1.21 (−1.71 to −0.71)	<0.001
Total propofol per anesthesia-hour (mg/kg/h)	5.23 ± 1.81 (*n* = 62)	3.59 ± 0.88 (*n* = 65)	−1.64 (−2.15 to −1.13)	<0.001
Remifentanil (mg)	1.65 ± 0.60 (*n* = 62)	1.63 ± 0.79 (*n* = 65)	−0.02 (−0.26 to 0.23)	0.878
Remifentanil per anesthesia-minute (µg/kg/min)	0.20 ± 0.05 (*n* = 62)	0.20 ± 0.06 (*n* = 65)	0.00 (−0.02 to 0.02)	0.962

Values are mean ± SD, with the observed denominator in parentheses. Mean difference (MD) is PS minus P; CI, confidence interval. Missing values were not imputed. *P* values are from Welch tests and are exploratory. Normalized maintenance exposure uses total recorded anesthesia time, not the maintenance-phase duration. One P induction amount is missing. One P total (320 mg) differs from its phase sum (370 mg); recorded totals are retained and sensitivity results are in Supplementary Table S5. Recorded surgery duration is not always identical to the interval between incision and surgery-end timestamps.

### 3.2 Induction hemodynamics

The primary MAP group-by-time interaction was not significant (ML random-intercept model: *χ*^2^ = 10.10, df = 12, *P* = 0.607; Figure 2A). The unstructured-covariance analysis gave a consistent result (*P* = 0.719). The cLDA-adjusted average post-baseline difference was 1.7 mmHg (95% CI, −2.0–5.5; *P* = 0.371). Nadir MAP averaged 69.8 ± 14.0 versus 72.8 ± 14.1 mmHg, a difference of 3.0 mmHg (95% CI, −1.9–8.0). Supportive outcomes appear in Table 3; landmark summaries are provided in Supplementary Tables S1A–S1D.

**Figure 2:**
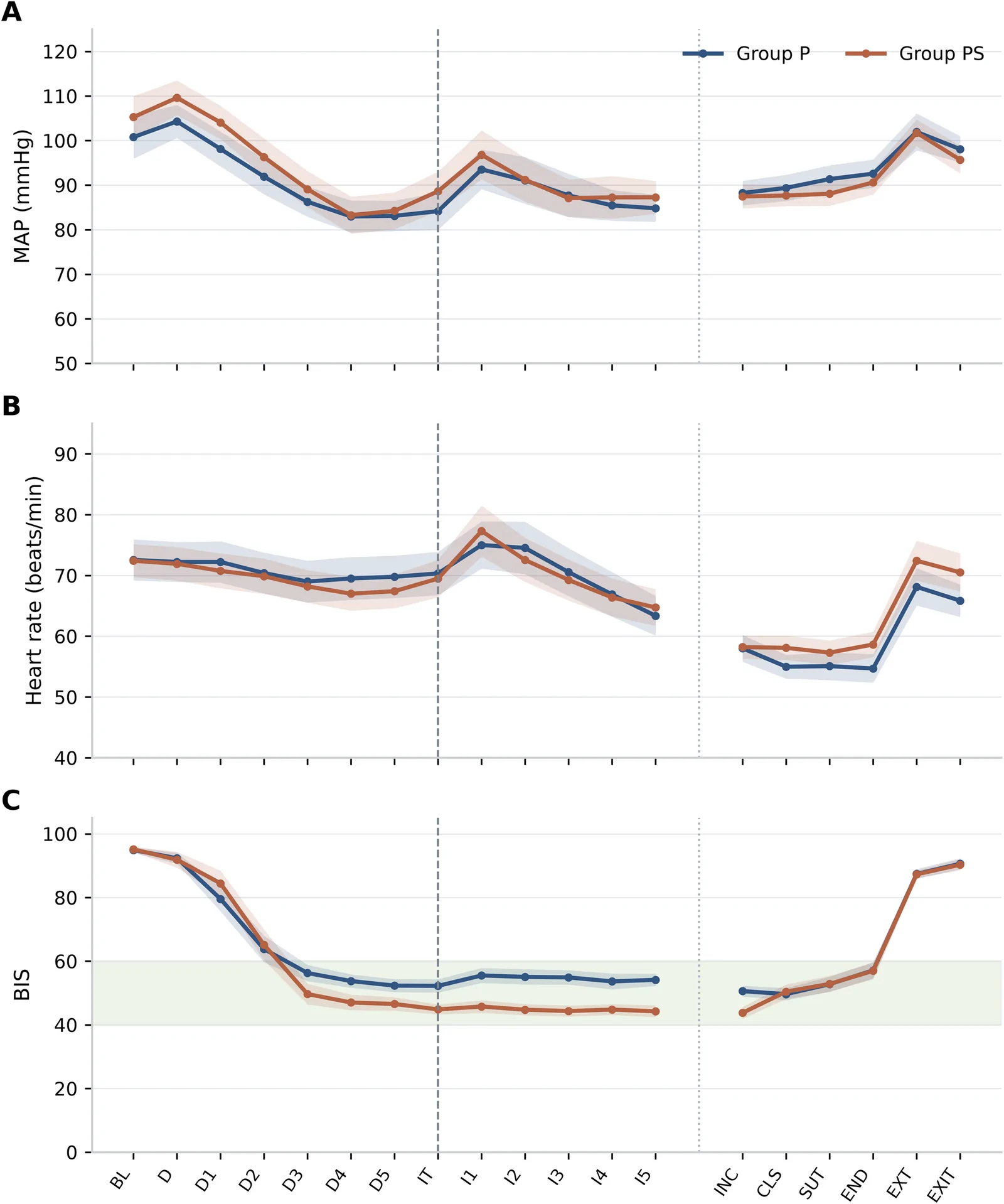
Recorded mean arterial pressure (MAP; **A**), heart rate **(B)**, and bispectral index (BIS; **C**) across 19 common landmarks. Points and lines show means; shaded ribbons show pointwise 95% t confidence intervals. The dashed vertical line marks intubation. The horizontal scale is categorical and does not represent equal elapsed time; a gap separates induction from later events. The ambiguous turning landmark is omitted. D, drug administration; D1–D5, nominal minutes after administration; IT, intubation; I1–I5, nominal minutes after intubation; INC, incision; CLS, wound closing; SUT, skin suturing; END, surgery end; EXT, extubation; EXIT, recorded exit landmark of unspecified location. Panel A starts at 50 mmHg and panel B at 40 beats/min; these truncated axes emphasize variation. The BIS target band is 40–60. MAP n = 62/65 except PS at END (n = 64); heart rate and BIS n = 62/65 throughout.

**Table 3:** Induction hemodynamic outcomes.

Outcome	Group P	Group PS	MD (95% CI)	*P* value
Nadir MAP (mmHg)	69.8 ± 14.0 (*n* = 62)	72.8 ± 14.1 (*n* = 65)	3.0 (−1.9 to 8.0)	0.226
Nadir SBP (mmHg)	105.0 ± 15.4 (*n* = 62)	110.0 ± 21.4 (*n* = 65)	5.0 (−1.5 to 11.6)	0.128
Maximum MAP change (mmHg)	−31.0 ± 19.0 (*n* = 62)	−32.4 ± 18.3 (*n* = 65)	−1.4 (−8.0 to 5.1)	0.663
Maximum MAP change (%)	−29.0 ± 16.4 (*n* = 62)	−29.5 ± 14.1 (*n* = 65)	−0.5 (−5.9 to 4.9)	0.867
Intubation MAP response (mmHg)	20.9 ± 17.6 (*n* = 62)	20.0 ± 20.0 (*n* = 65)	−0.9 (−7.5 to 5.7)	0.780
Peak post-intubation MAP (mmHg)	104.0 ± 18.4 (*n* = 62)	104.2 ± 22.3 (*n* = 65)	0.2 (−7.0 to 7.3)	0.960
Readings with MAP <65, median [IQR]	0.0 [0.0–1.0] (*n* = 62)	0.0 [0.0–0.0] (*n* = 65)	−0.1 (−0.5 to 0.3)	0.368
Clock-based area <65 (mmHg·min), median [IQR]	0.00 [0.00–2.22] (*n* = 54)	0.00 [0.00–0.05] (*n* = 63)	0.00 (−4.73 to 4.72)	0.320

Binary outcomes.					
Outcome	Group P	Group PS	RD, pp (95% CI)	RR (95% CI)	*P* value
SBP-defined hypotension	37/62 (59.7%)	32/65 (49.2%)	−10.4 (−26.8 to 6.7)	0.82 (0.60–1.14)	0.286
Any MAP <65 mmHg	19/62 (30.6%)	16/65 (24.6%)	−6.0 (−21.2 to 9.4)	0.80 (0.46–1.42)	0.552
Any MAP <60 mmHg	10/62 (16.1%)	8/65 (12.3%)	−3.8 (−16.4 to 8.6)	0.76 (0.32–1.81)	0.615
Any MAP <55 mmHg	5/62 (8.1%)	6/65 (9.2%)	1.2 (−9.5 to 11.7)	1.14 (0.37–3.56)	1.000
Any MAP decrease ≥20%	48/62 (77.4%)	52/65 (80.0%)	2.6 (−11.6 to 16.8)	1.03 (0.86–1.24)	0.829

Induction summaries include the 13 landmarks from baseline through 5 min after intubation. Protocol hypotension is SBP <80 mmHg or a ≥ 30% SBP decrease. Continuous rows show mean ± SD unless labeled median [IQR]; MD and its CI always estimate differences in means. Count/area *P* values use Mann–Whitney tests, while other continuous *P* values use Welch tests. Area is exploratory, requires strictly increasing clock times, and assumes linear MAP between readings. Binary *P* values use Fisher exact tests; RD, risk difference (PS minus P) in percentage points (pp); RR, risk ratio (PS/P); CIs use Newcombe and log-scale methods, respectively. No equivalence margin was specified.

SBP-defined hypotension occurred in 37/62 versus 32/65 patients (59.7% versus 49.2%; Figure 3). The risk ratio was 0.82 (95% CI, 0.60–1.14), and the risk difference was −10.4 percentage points (95% CI, −26.8–6.7). Any MAP <65 mmHg occurred in 19/62 versus 16/65 patients. Threshold counts and the reconstructed area had medians of zero in both groups (Table 3). The clock-based area analysis included 54 P and 63 PS patients. Taken together, the pressure outcomes did not establish superiority, and their CIs do not establish equivalence.

**Figure 3:**
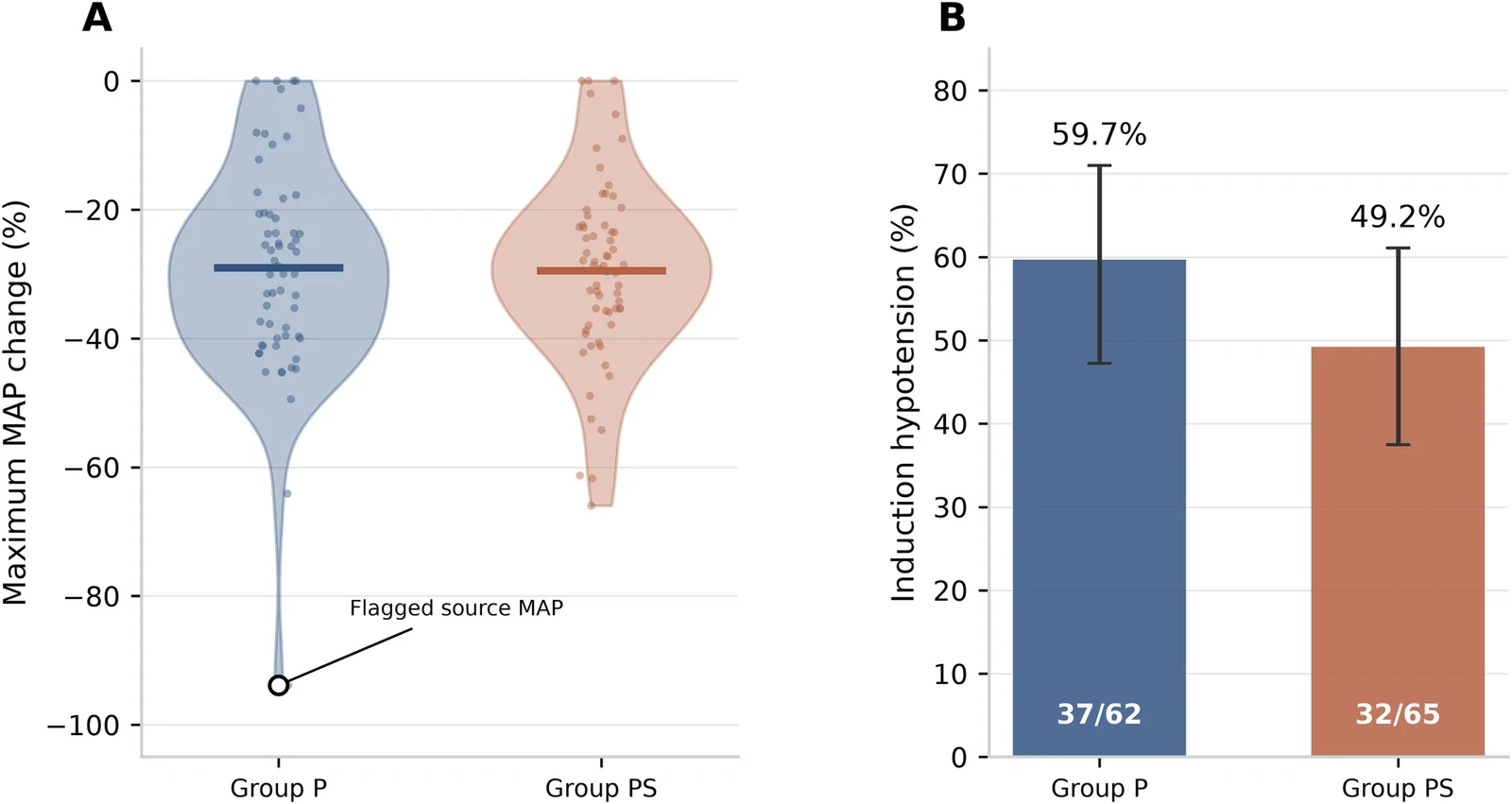
Maximum percentage MAP change from the preinduction baseline **(A)** and SBP-defined induction hypotension **(B)**. In A, violins show kernel density estimates, dots show all patients, and horizontal lines show group means. Densities are clipped to the observed range. The open marker identifies the retained MAP value of 6 mmHg that is inconsistent with its paired BP. In B, hypotension means any SBP <80 mmHg or a ≥ 30% SBP decrease over the 13 induction landmarks. Bars show proportions and error bars are Wilson 95% confidence intervals; labels show percentages and counts/denominators.

Induction notes documented at least one vasoactive agent in 17/62 P and 12/65 PS patients. Counts for norepinephrine, methoxamine, dopamine, and epinephrine were 2 versus 5, 9 versus 6, 11 versus 7, and 0 versus 1, respectively. Agents overlapped within patients; atropine was documented separately in one PS patient. These are minimum documented counts rather than complete treatment incidences (Supplementary Table S3). The MAP comparison therefore describes the assigned strategies under clinical rescue management.

### 3.3 BIS and heart-rate trajectories

BIS trajectories differed during induction (ML interaction *P* < 0.001; Figure 2C). At intubation, BIS averaged 52.2 ± 8.3 versus 44.8 ± 6.2, a difference of −7.4 (95% CI, −10.0 to −4.8). BIS <40 during 1–5 min after intubation occurred in 2/62 versus 22/65 patients (risk difference, 30.6 percentage points; 95% CI, 17.7–43.0). At the four maintenance landmarks, corresponding counts were 6/62 versus 20/65 (difference, 21.1 percentage points; 95% CI, 7.1–34.2). Table 4 and Figure 4 present these distinct windows.

**Table 4:** BIS outcomes.

Outcome	Group P	Group PS	MD (95% CI)	*P* value
BIS at intubation	52.2 ± 8.3 (*n* = 62)	44.8 ± 6.2 (*n* = 65)	−7.4 (−10.0 to −4.8)	<0.001
BIS 1 min after intubation	55.5 ± 9.2 (*n* = 62)	45.8 ± 7.9 (*n* = 65)	−9.8 (−12.8 to −6.7)	<0.001
BIS at incision	50.6 ± 6.6 (*n* = 62)	43.8 ± 7.5 (*n* = 65)	−6.8 (−9.3 to −4.3)	<0.001
Mean maintenance BIS	52.5 ± 7.0 (*n* = 62)	51.0 ± 7.4 (*n* = 65)	−1.5 (−4.0 to 1.0)	0.236
Within-patient maintenance BIS SD	5.3 ± 3.5 (*n* = 62)	7.3 ± 4.6 (*n* = 65)	2.0 (0.6 to 3.4)	0.007
Maintenance readings outside 40–60 (%)	25.0 ± 28.6 (*n* = 62)	24.2 ± 26.1 (*n* = 65)	−0.8 (−10.4 to 8.9)	0.875
BIS at extubation	87.4 ± 6.1 (*n* = 62)	87.3 ± 4.9 (*n* = 65)	−0.1 (−2.1 to 1.8)	0.898
BIS at recorded exit	90.6 ± 6.8 (*n* = 62)	90.3 ± 7.0 (*n* = 65)	−0.3 (−2.7 to 2.1)	0.814

Binary outcomes					
Outcome	Group P	Group PS	RD, pp (95% CI)	RR (95% CI)	*P* value
Any BIS <40, 1–5 min after intubation	2/62 (3.2%)	22/65 (33.8%)	30.6 (17.7 to 43.0)	10.49 (2.57–42.77)	<0.001
Any BIS <40 during maintenance	6/62 (9.7%)	20/65 (30.8%)	21.1 (7.1 to 34.2)	3.18 (1.37–7.39)	0.004

BIS is a processed EEG index. Continuous rows show mean ± SD and available n; between-group CIs are Welch intervals. Maintenance comprises four landmarks: incision, closing, suturing, and surgery end. The post-intubation window comprises five readings at 1–5 min, excluding the intubation reading. Binary CIs and tests follow Table 3. A BIS threshold alone does not establish equivalent or excessive hypnotic depth across drug regimens.

**Figure 4:**
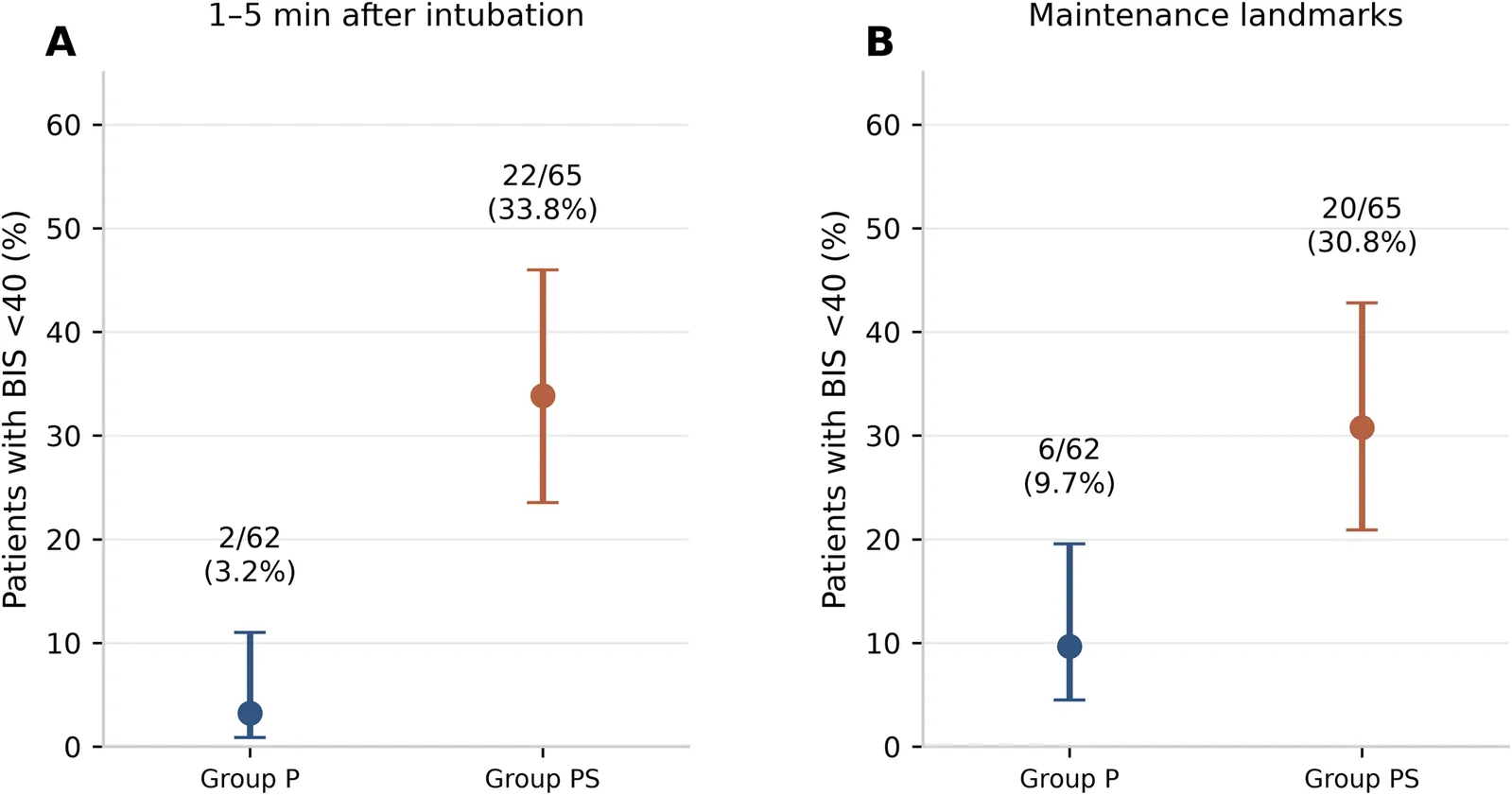
Patients with at least one recorded BIS <40 in two distinct windows: 1–5 min after intubation **(A)** and the four maintenance landmarks from incision to surgery end **(B)**. Points show proportions and error bars show Wilson 95% confidence intervals. Labels show events/participants. These are recorded BIS thresholds, not independently validated diagnoses of excessive hypnosis.

Across 19 common landmarks, exploratory GEE interaction tests gave *P* = 0.092 for MAP, *P* = 0.030 for heart rate, and *P* < 0.001 for BIS. Heart-rate differences varied across emergence landmarks. The extubation contrast was 4.3 beats/min (95% CI, −0.1–8.7), compared with 4.7 beats/min at recorded exit (95% CI, 0.6–8.7). Full landmark summaries, including PPV, appear in Supplementary Tables S1A–S1D.

### 3.4 Propofol exposure and perioperative measures

Recorded total propofol use was 31.4% lower in PS: 751.3 ± 412.2 versus 515.4 ± 319.3 mg (difference, −235.9 mg; 95% CI, −366.0 to −105.9). Maintenance amounts were also lower, at 618.5 ± 401.9 versus 445.0 ± 319.0 mg (difference, −173.5 mg; 95% CI, −301.5 to −45.6). The contrast persisted after normalization to body weight and total anesthesia duration (Table 2; Figure 5). Maintenance dosing was guided by visible BIS. One inconsistent phase/total record was retained; excluding it or substituting its arithmetic phase sum preserved the direction of the contrast (Supplementary Table S5). Remifentanil totals are shown in Table 2; sufentanil exposure was unavailable.

**Figure 5:**
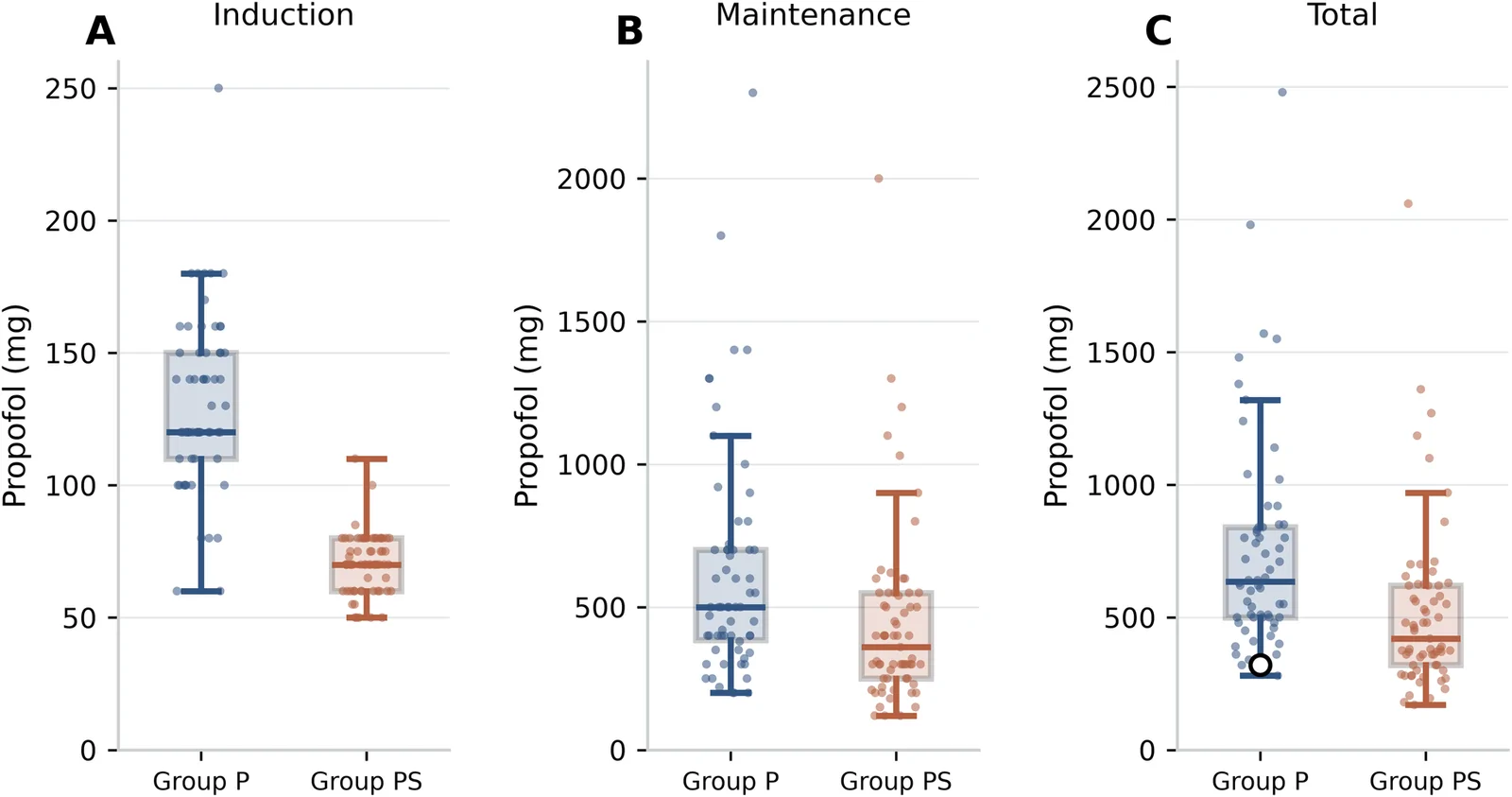
Recorded propofol amounts during induction **(A)**, maintenance **(B)**, and in total **(C)**. Boxes show medians and interquartile ranges; whiskers extend to the most extreme observations within 1.5 interquartile ranges. Dots show all observed patient values with deterministic horizontal jitter. P induction *n* = 61; all other group-phase combinations *n* = 62 for P and 65 for PS. One missing induction amount is not reconstructed. The open marker in C denotes the retained inconsistent total of 320 mg for a P patient whose recorded phase amounts sum to 370 mg. Colors consistently identify treatment groups, not phases.

Net fluid balance averaged 796.9 ± 306.4 versus 914.8 ± 336.6 mL (difference, 117.9 mL; 95% CI, 5.0–230.9; Supplementary Table S4). Fluid timing was unavailable. Missing component entries were not assumed to represent zero exposure.

Baseline potassium was <3.5 mmol/L in 13/61 P and 15/65 PS patients. Extubation values were available for 58 and 62 patients, with complete baseline–extubation pairs in 57 and 62. The between-group difference in mean paired change was 0.10 mmol/L (95% CI, −0.06–0.26; Table 5). One PS patient received documented potassium supplementation, with values of 3.0 mmol/L at baseline and 4.0 mmol/L at extubation.

**Table 5:** Recorded recovery, potassium, and postoperative outcomes.

Outcome	Group P	Group PS	MD (95% CI)	*P* value
Surgery end to extubation (min)	10.5 ± 4.2 (*n* = 62)	13.6 ± 7.8 (*n* = 65)	3.1 (0.9 to 5.2)	0.007
MAP at extubation (mmHg)	102.0 ± 16.3 (*n* = 62)	101.8 ± 12.2 (*n* = 65)	−0.2 (−5.3 to 4.9)	0.939
Heart rate at extubation (beats/min)	68.1 ± 12.0 (*n* = 62)	72.4 ± 13.2 (*n* = 65)	4.3 (−0.1 to 8.7)	0.057
MAP at recorded exit (mmHg)	98.1 ± 11.5 (*n* = 62)	95.7 ± 12.5 (*n* = 65)	−2.4 (−6.6 to 1.8)	0.267
Heart rate at recorded exit (beats/min)	65.9 ± 10.5 (*n* = 62)	70.5 ± 12.7 (*n* = 65)	4.7 (0.6 to 8.7)	0.026
MAP change, baseline to recorded exit (mmHg)	−2.7 ± 18.7 (*n* = 62)	−9.6 ± 18.9 (*n* = 65)	−6.9 (−13.5 to −0.3)	0.042
Extubation potassium (mmol/L)	3.79 ± 0.41 (*n* = 58)	3.77 ± 0.30 (*n* = 62)	−0.02 (−0.15 to 0.11)	0.757
Paired potassium change (mmol/L)	0.09 ± 0.53 (*n* = 57)	0.19 ± 0.33 (*n* = 62)	0.10 (−0.06 to 0.26)	0.229
NRS, postoperative day 1	1.98 ± 1.17 (*n* = 62)	1.82 ± 1.40 (*n* = 65)	−0.17 (−0.62 to 0.28)	0.462
NRS, postoperative day 2	1.24 ± 1.60 (*n* = 62)	0.88 ± 1.01 (*n* = 65)	−0.37 (−0.84 to 0.11)	0.128
NRS, postoperative day 3	1.21 ± 1.55 (*n* = 62)	0.75 ± 1.09 (*n* = 65)	−0.46 (−0.93 to 0.02)	0.059

Binary outcomes.					
Outcome	Group P	Group PS	RD, pp (95% CI)	RR (95% CI)	*P* value
Extubation SBP ≥180 mmHg	4/62 (6.5%)	6/65 (9.2%)	2.8 (−7.5 to 13.0)	1.43 (0.42–4.83)	0.744
Recorded delirium	1/62 (1.6%)	0/65 (0.0%)	−1.6 (−8.6 to 4.1)	Not estimable	0.488
Recorded PONV	10/62 (16.1%)	10/65 (15.4%)	−0.7 (−13.7 to 12.1)	0.95 (0.43–2.13)	1.000

Continuous rows show mean ± SD and observed n; *P* values use Welch tests. All comparisons are exploratory. Surgery-end-to-extubation medians were 10 (8–13) versus 10 (8–15) min (Mann–Whitney *P* = 0.074); this distributional test differs from a test of mean intervals. Potassium changes use complete baseline/extubation pairs. PONV, postoperative nausea and vomiting; NRS, numeric rating scale. Delirium/PONV instruments and observation windows were not verified. The exit location was unspecified. An RR confidence interval is not estimated by the conventional log method when one group has no events.

### 3.5 Extubation interval and postoperative outcomes

The surgery-end-to-extubation interval averaged 10.5 ± 4.2 versus 13.6 ± 7.8 min (difference, 3.1 min; 95% CI, 0.9–5.2; Welch *P* = 0.007). Both medians were 10 min, with interquartile ranges of 8–13 and 8–15 min (Mann–Whitney *P* = 0.074; Figure 6A). The mean and distributional comparisons address different questions. This exploratory interval describes the timing of recorded extubation, rather than the timing of regained consciousness.

**Figure 6:**
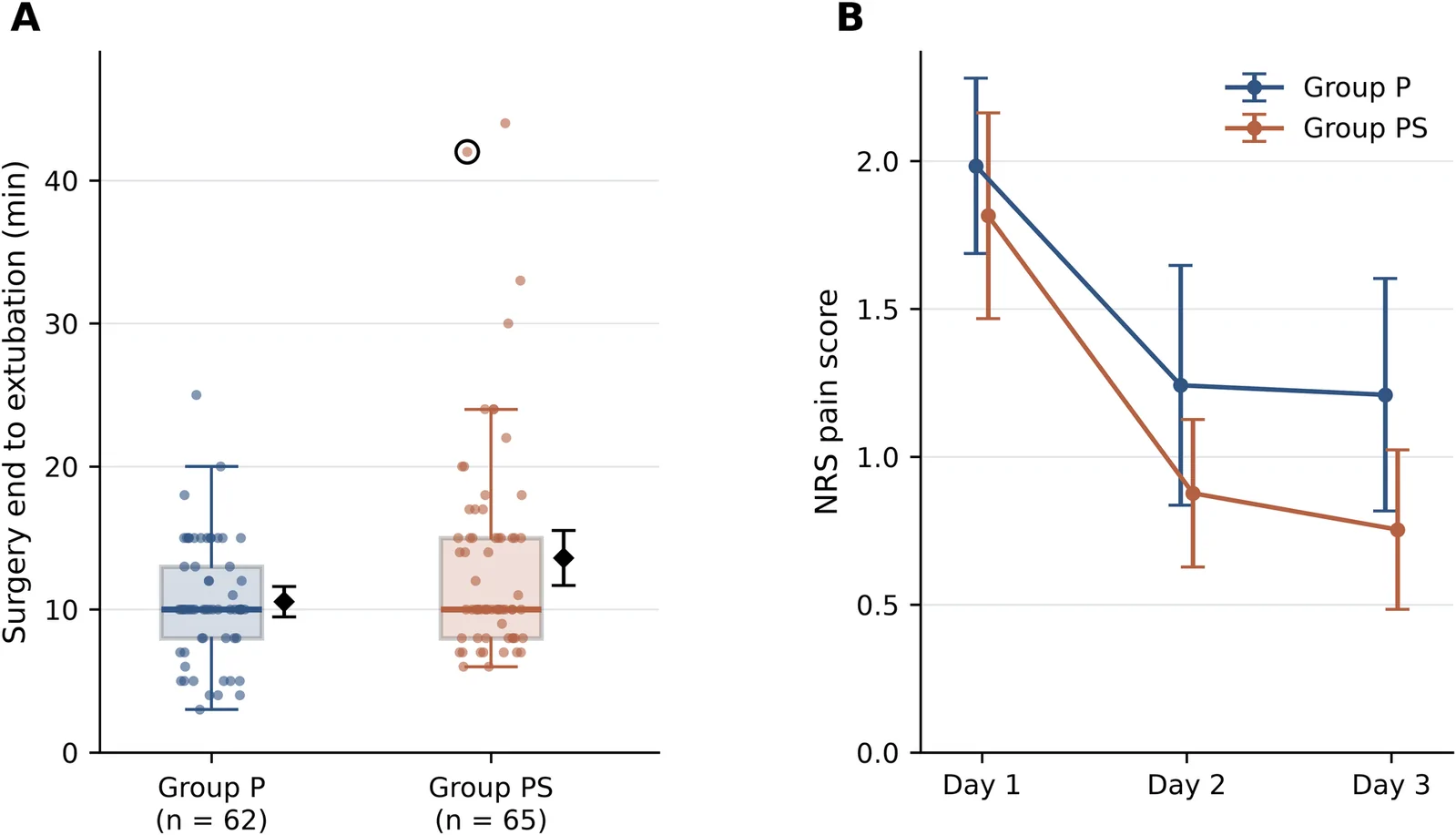
Recorded extubation interval and postoperative pain. **(A)** Surgery-end-to-extubation intervals for all patients. Boxes show medians and interquartile ranges; whiskers extend to observations within 1.5 interquartile ranges. Dots show individual records; black diamonds and error bars show means and pointwise 95% t confidence intervals. The open circle flags the PS patient whose recorded exit preceded extubation; this record remains in the main analysis. **(B)** NRS pain scores on postoperative days 1–3, shown as means with pointwise 95% t confidence intervals. P *n* = 62 and PS *n* = 65 in both panels. Pain assessment at rest versus movement was unspecified. All comparisons are exploratory.

NRS scores were low on postoperative days 1–3 (Table 5; Figure 6B). Recorded delirium occurred in 1/62 versus 0/65 patients (Fisher *P* = 0.488), and PONV in 10/62 versus 10/65. These outcomes provide descriptive observations of the early postoperative course; limited event counts and undocumented assessment procedures constrain comparisons.

### 3.6 Sensitivity analyses

A MAP value of 6 mmHg was paired with BP 105/51 mmHg at intubation in one P patient. Across induction, 29 MAP readings in 25 patients lay outside their paired SBP–diastolic BP range; 20 readings in 19 patients exceeded it by >5 mmHg. GEE interaction *P* values were 0.699 with all readings, 0.830 after strict masking, and 0.729 with the 5-mmHg rule. Restricting the analysis to the 120 patients aged 40–80 years gave *P* = 0.549. These analyses did not change the interpretation of the primary MAP result (Supplementary Tables S2 and S5).

## 4 Discussion

This randomized trial shows that the reduction in propofol use with an oxybate-containing induction strategy extended into maintenance during elective spine surgery. Lower BIS values and a longer mean extubation interval accompanied the reduction, while the primary analysis did not demonstrate hemodynamic superiority. Together, these findings characterize how the combined regimen performed under BIS-guided care. They extend earlier sedation evidence to a setting in which co-administered drugs and clinical titration jointly shape anesthetic exposure and recovery.

The BIS findings are central to that interpretation. Sodium oxybate alters EEG activity (11), creating a plausible drug-specific explanation for lower processed index values. In 36 children receiving GHB for burn care, BIS tracked clinical sedation (8). That finding supports sensitivity to sedation in that setting, but does not validate a BIS threshold of 40 during adult propofol–oxybate anesthesia. Greater hypnosis and altered index calibration may both contribute; the present recordings cannot distinguish them. If BIS overstates hypnosis, titration to a conventional target could permit more propofol reduction than an independent assessment would justify. Raw EEG, suppression measures, signal quality, and structured awareness assessment are therefore needed to evaluate this strategy.

The reduction in propofol use extended beyond the planned induction-dose difference into the maintenance phase. It also remained evident after normalization to body weight and total anesthesia duration. These observations characterize the assigned strategy in clinical use. Because maintenance dosing responded to visible BIS, the reduction integrates the adjunct's effects with clinicians' titration decisions. It cannot be attributed entirely to pharmacological substitution at matched hypnotic depth. Similar remifentanil totals provide additional context, although unavailable sufentanil doses leave co-medication exposure incompletely characterized.

The extubation data add a further dimension to this profile. Both groups had a median interval of 10 min, but PS had a longer mean and greater dispersion (Figure 6A). These summaries describe different aspects of the same distribution and should be interpreted together. The interval also includes unblinded clinical decisions and is not a direct measure of regained consciousness. It is therefore best viewed as a recovery-related observation that can guide the choice of endpoints in subsequent trials.

Reducing propofol alone does not reliably eliminate induction hypotension, as illustrated by a randomized dose-comparison trial (12). In this study, neither trajectory models nor baseline-adjusted estimates demonstrated a MAP advantage. Rescue treatment, fluid administration, and a single preinduction baseline reading complicate interpretation of the pressure response. Missing rescue doses prevent separation of intrinsic drug effects from clinical compensation. Etomidate remains an established induction alternative with relatively limited cardiovascular depression, balanced against myoclonus and adrenal steroid-synthesis suppression (13). The present comparison does not establish an advantage over etomidate. It instead addresses whether oxybate can usefully supplement an existing propofol-based strategy.

The pragmatic sample size was not supported by prospective power planning. The baseline-adjusted average contrast across 12 post-baseline landmarks was 1.7 mmHg (95% CI, −2.0–5.5), whereas the contrast between patient-specific nadirs was 3.0 mmHg (95% CI, −1.9 to 8.0). These estimates describe different aspects of the pressure response. Their CIs encompass no difference and effects in either direction, leaving clinically relevant benefit unresolved. The intervals quantify precision; they neither establish equivalence nor retrospectively justify the enrollment target. The nonsignificant primary result therefore leaves the hemodynamic question open.

Formulation and exposure also matter. European product information describes oral sodium oxybate for narcolepsy (14); it does not establish indications for the intravenous product studied here. The mean 2.06-g dose contributed approximately 376 mg (16.3 mmol) of sodium from the active salt, excluding any excipient sodium. A study in narcolepsy linked switching from high- to low-sodium oxybate to blood-pressure changes over several weeks (15). That chronic oral exposure differs from a single intravenous dose, but provides a rationale for reporting sodium burden explicitly.

Position and monitoring further define the setting in which these results apply. Position-related hypotension has been described in spine surgery (16), but the current recordings do not isolate the transition to prone positioning. A randomized trial also showed that monitoring strategy can affect hypotension detection and management (17). The scheduled landmark readings cannot establish the duration of hypotension between observations. The findings therefore apply to sampled hemodynamics across heterogeneous spine procedures, without a position-specific efficacy claim.

The principal limitations concern treatment documentation, allocation concealment, and outcome ascertainment. Dose discrepancies and unavailable administration details limit reproducibility; missing screening logs limit assessment of eligibility deviations. Internal BP and timestamp inconsistencies remain despite sensitivity analyses. Available-case potassium and area analyses may be affected by selective missingness, and multiple exploratory comparisons increase the risk of chance findings. Sparse adverse-event records, absent early potassium sampling, and no structured awareness assessment preclude a comprehensive safety evaluation. A subsequent trial should standardize administration and rescue treatment, document allocation concealment, and pair EEG measures with independently assessed hypnosis and recovery.

## 5 Conclusions

In this randomized trial of elective spine surgery, sodium oxybate with reduced-dose propofol was associated with approximately one-third lower total propofol use, including lower maintenance use. Lower BIS values and a longer mean extubation interval accompanied this reduction. Hemodynamic superiority was not demonstrated. These findings define the observed effects and trade-offs of the combined regimen under BIS-guided care and inform further evaluation of dose selection, hypnosis monitoring, and recovery.

## Data Availability

The original contributions presented in the study are included in the article/Supplementary Material, further inquiries can be directed to the corresponding author/s.
