# Supplementary material for: Sodium oxybate with reduced-dose propofol for anesthesia induction in elective spine surgery: a randomized trial
Source: Front Med (Lausanne). 2026 Oct 13;13:1970835. doi: 10.3389/fmed.2026.1970835 (PMC13710391; doi:10.3389/fmed.2026.1970835)
Supplement: Supplementary file 1 [file Table1.docx]

Supplementary Material

## Table S1A. MAP (mmHg) at the 19 common landmarks

| **Outcome** | **Group P** | **Group PS** | **MD (95% CI)** | **P value** |
| --- | --- | --- | --- | --- |
| Baseline | 100.8 ± 19.0 (n = 62) | 105.3 ± 18.9 (n = 65) | — | — |
| Drug administration | 104.3 ± 14.6 (n = 62) | 109.6 ± 15.7 (n = 65) | 5.3 (0.0 to 10.6) | 0.050 |
| Drug +1 min | 98.1 ± 15.4 (n = 62) | 104.1 ± 15.0 (n = 65) | 5.9 (0.6 to 11.3) | 0.030 |
| Drug +2 min | 91.9 ± 15.6 (n = 62) | 96.3 ± 16.9 (n = 65) | 4.4 (−1.3 to 10.1) | 0.130 |
| Drug +3 min | 86.3 ± 13.3 (n = 62) | 89.1 ± 16.3 (n = 65) | 2.8 (−2.4 to 8.0) | 0.288 |
| Drug +4 min | 83.0 ± 14.2 (n = 62) | 83.3 ± 16.7 (n = 65) | 0.3 (−5.1 to 5.7) | 0.911 |
| Drug +5 min | 83.1 ± 13.5 (n = 62) | 84.2 ± 16.6 (n = 65) | 1.1 (−4.2 to 6.4) | 0.677 |
| Intubation | 84.2 ± 16.4 (n = 62) | 88.6 ± 17.6 (n = 65) | 4.5 (−1.5 to 10.5) | 0.142 |
| Intubation +1 min | 93.5 ± 17.4 (n = 62) | 96.8 ± 22.2 (n = 65) | 3.3 (−3.7 to 10.3) | 0.357 |
| Intubation +2 min | 91.1 ± 20.7 (n = 62) | 91.2 ± 19.8 (n = 65) | 0.1 (−7.0 to 7.3) | 0.970 |
| Intubation +3 min | 87.7 ± 19.2 (n = 62) | 87.1 ± 17.0 (n = 65) | −0.6 (−7.0 to 5.8) | 0.852 |
| Intubation +4 min | 85.5 ± 13.6 (n = 62) | 87.3 ± 19.3 (n = 65) | 1.8 (−4.1 to 7.6) | 0.545 |
| Intubation +5 min | 84.8 ± 12.0 (n = 62) | 87.3 ± 14.8 (n = 65) | 2.5 (−2.3 to 7.2) | 0.303 |
| Skin incision | 88.2 ± 10.8 (n = 62) | 87.5 ± 10.9 (n = 65) | −0.8 (−4.6 to 3.0) | 0.686 |
| Wound closing | 89.4 ± 11.5 (n = 62) | 87.7 ± 9.5 (n = 65) | −1.7 (−5.4 to 2.0) | 0.365 |
| Skin suturing | 91.4 ± 12.1 (n = 62) | 88.1 ± 10.9 (n = 65) | −3.3 (−7.4 to 0.8) | 0.111 |
| End of surgery | 92.6 ± 12.5 (n = 62) | 90.6 ± 10.8 (n = 64) | −2.0 (−6.1 to 2.2) | 0.346 |
| Extubation | 102.0 ± 16.3 (n = 62) | 101.8 ± 12.2 (n = 65) | −0.2 (−5.3 to 4.9) | 0.939 |
| Recorded exit | 98.1 ± 11.5 (n = 62) | 95.7 ± 12.5 (n = 65) | −2.4 (−6.6 to 1.8) | 0.267 |

Group summaries are mean ± SD with observed n. Baseline values are descriptive; no baseline contrasts or P values are presented. Post-baseline mean differences and 95% CIs use Welch calculations; P values are unadjusted pointwise descriptions. Landmarks are categorical, not equally spaced elapsed-time observations. The turning landmark is omitted because its meaning differs or is unverified across patients. “Recorded exit” does not imply verified PACU discharge.

## Table S1B. Heart rate (beats/min) at the 19 common landmarks

| **Outcome** | **Group P** | **Group PS** | **MD (95% CI)** | **P value** |
| --- | --- | --- | --- | --- |
| Baseline | 72.6 ± 13.3 (n = 62) | 72.4 ± 11.0 (n = 65) | — | — |
| Drug administration | 72.2 ± 12.9 (n = 62) | 71.9 ± 11.1 (n = 65) | −0.3 (−4.5 to 3.9) | 0.888 |
| Drug +1 min | 72.2 ± 13.4 (n = 62) | 70.8 ± 11.6 (n = 65) | −1.5 (−5.9 to 2.9) | 0.511 |
| Drug +2 min | 70.4 ± 13.2 (n = 62) | 69.9 ± 11.7 (n = 65) | −0.5 (−4.9 to 3.9) | 0.813 |
| Drug +3 min | 69.0 ± 13.5 (n = 62) | 68.2 ± 10.8 (n = 65) | −0.8 (−5.1 to 3.5) | 0.714 |
| Drug +4 min | 69.5 ± 13.9 (n = 62) | 67.0 ± 11.3 (n = 65) | −2.5 (−7.0 to 1.9) | 0.266 |
| Drug +5 min | 69.8 ± 13.7 (n = 62) | 67.4 ± 11.3 (n = 65) | −2.4 (−6.8 to 2.1) | 0.290 |
| Intubation | 70.3 ± 14.0 (n = 62) | 69.5 ± 12.5 (n = 65) | −0.8 (−5.5 to 3.8) | 0.720 |
| Intubation +1 min | 75.0 ± 15.3 (n = 62) | 77.3 ± 16.9 (n = 65) | 2.3 (−3.4 to 7.9) | 0.424 |
| Intubation +2 min | 74.5 ± 17.0 (n = 62) | 72.6 ± 14.6 (n = 65) | −2.0 (−7.6 to 3.6) | 0.484 |
| Intubation +3 min | 70.5 ± 15.5 (n = 62) | 69.3 ± 13.6 (n = 65) | −1.3 (−6.4 to 3.9) | 0.621 |
| Intubation +4 min | 66.9 ± 14.5 (n = 62) | 66.4 ± 12.6 (n = 65) | −0.5 (−5.3 to 4.3) | 0.835 |
| Intubation +5 min | 63.4 ± 12.8 (n = 62) | 64.7 ± 12.2 (n = 65) | 1.4 (−3.0 to 5.8) | 0.539 |
| Skin incision | 58.0 ± 8.7 (n = 62) | 58.2 ± 7.7 (n = 65) | 0.2 (−2.7 to 3.1) | 0.892 |
| Wound closing | 55.0 ± 7.7 (n = 62) | 58.1 ± 8.1 (n = 65) | 3.1 (0.3 to 5.9) | 0.029 |
| Skin suturing | 55.1 ± 9.0 (n = 62) | 57.3 ± 8.1 (n = 65) | 2.2 (−0.8 to 5.2) | 0.147 |
| End of surgery | 54.7 ± 9.2 (n = 62) | 58.6 ± 8.5 (n = 65) | 4.0 (0.8 to 7.1) | 0.013 |
| Extubation | 68.1 ± 12.0 (n = 62) | 72.4 ± 13.2 (n = 65) | 4.3 (−0.1 to 8.7) | 0.057 |
| Recorded exit | 65.9 ± 10.5 (n = 62) | 70.5 ± 12.7 (n = 65) | 4.7 (0.6 to 8.7) | 0.026 |

Group summaries are mean ± SD with observed n. Baseline values are descriptive; no baseline contrasts or P values are presented. Post-baseline mean differences and 95% CIs use Welch calculations; P values are unadjusted pointwise descriptions. Landmarks are categorical, not equally spaced elapsed-time observations. The turning landmark is omitted because its meaning differs or is unverified across patients. “Recorded exit” does not imply verified PACU discharge.

## Table S1C. BIS at the 19 common landmarks

| **Outcome** | **Group P** | **Group PS** | **MD (95% CI)** | **P value** |
| --- | --- | --- | --- | --- |
| Baseline | 94.9 ± 3.5 (n = 62) | 95.1 ± 3.3 (n = 65) | — | — |
| Drug administration | 92.3 ± 6.8 (n = 62) | 91.9 ± 10.1 (n = 65) | −0.5 (−3.5 to 2.5) | 0.762 |
| Drug +1 min | 79.6 ± 15.4 (n = 62) | 84.4 ± 16.2 (n = 65) | 4.9 (−0.7 to 10.4) | 0.084 |
| Drug +2 min | 63.8 ± 16.4 (n = 62) | 65.1 ± 19.5 (n = 65) | 1.3 (−5.0 to 7.6) | 0.681 |
| Drug +3 min | 56.3 ± 10.0 (n = 62) | 49.7 ± 13.1 (n = 65) | −6.6 (−10.7 to −2.5) | 0.002 |
| Drug +4 min | 53.8 ± 8.3 (n = 62) | 47.0 ± 9.9 (n = 65) | −6.7 (−9.9 to −3.5) | <0.001 |
| Drug +5 min | 52.3 ± 8.0 (n = 62) | 46.6 ± 8.6 (n = 65) | −5.8 (−8.7 to −2.9) | <0.001 |
| Intubation | 52.2 ± 8.3 (n = 62) | 44.8 ± 6.2 (n = 65) | −7.4 (−10.0 to −4.8) | <0.001 |
| Intubation +1 min | 55.5 ± 9.2 (n = 62) | 45.8 ± 7.9 (n = 65) | −9.8 (−12.8 to −6.7) | <0.001 |
| Intubation +2 min | 55.1 ± 9.4 (n = 62) | 44.8 ± 7.1 (n = 65) | −10.3 (−13.3 to −7.4) | <0.001 |
| Intubation +3 min | 54.9 ± 8.9 (n = 62) | 44.3 ± 6.9 (n = 65) | −10.6 (−13.4 to −7.7) | <0.001 |
| Intubation +4 min | 53.6 ± 9.8 (n = 62) | 44.8 ± 6.8 (n = 65) | −8.8 (−11.8 to −5.9) | <0.001 |
| Intubation +5 min | 54.1 ± 7.5 (n = 62) | 44.2 ± 7.4 (n = 65) | −9.9 (−12.5 to −7.3) | <0.001 |
| Skin incision | 50.6 ± 6.6 (n = 62) | 43.8 ± 7.5 (n = 65) | −6.8 (−9.3 to −4.3) | <0.001 |
| Wound closing | 49.6 ± 7.6 (n = 62) | 50.4 ± 9.2 (n = 65) | 0.8 (−2.2 to 3.7) | 0.599 |
| Skin suturing | 52.8 ± 9.2 (n = 62) | 52.9 ± 10.1 (n = 65) | 0.1 (−3.3 to 3.5) | 0.959 |
| End of surgery | 57.1 ± 10.0 (n = 62) | 57.0 ± 10.6 (n = 65) | −0.1 (−3.8 to 3.5) | 0.937 |
| Extubation | 87.4 ± 6.1 (n = 62) | 87.3 ± 4.9 (n = 65) | −0.1 (−2.1 to 1.8) | 0.898 |
| Recorded exit | 90.6 ± 6.8 (n = 62) | 90.3 ± 7.0 (n = 65) | −0.3 (−2.7 to 2.1) | 0.814 |

Group summaries are mean ± SD with observed n. Baseline values are descriptive; no baseline contrasts or P values are presented. Post-baseline mean differences and 95% CIs use Welch calculations; P values are unadjusted pointwise descriptions. Landmarks are categorical, not equally spaced elapsed-time observations. The turning landmark is omitted because its meaning differs or is unverified across patients. “Recorded exit” does not imply verified PACU discharge.

## Table S1D. PPV (%) at the 19 common landmarks

| **Outcome** | **Group P** | **Group PS** | **MD (95% CI)** | **P value** |
| --- | --- | --- | --- | --- |
| Baseline | 5.2 ± 1.7 (n = 62) | 5.4 ± 1.9 (n = 65) | — | — |
| Drug administration | 5.2 ± 1.6 (n = 62) | 5.1 ± 2.1 (n = 65) | −0.1 (−0.7 to 0.6) | 0.870 |
| Drug +1 min | 6.0 ± 1.9 (n = 62) | 5.6 ± 2.2 (n = 65) | −0.5 (−1.2 to 0.3) | 0.207 |
| Drug +2 min | 7.3 ± 2.5 (n = 62) | 7.0 ± 2.9 (n = 65) | −0.3 (−1.2 to 0.7) | 0.588 |
| Drug +3 min | 8.2 ± 3.1 (n = 62) | 7.8 ± 2.9 (n = 65) | −0.4 (−1.5 to 0.7) | 0.464 |
| Drug +4 min | 8.7 ± 2.5 (n = 62) | 9.0 ± 2.9 (n = 65) | 0.2 (−0.7 to 1.2) | 0.612 |
| Drug +5 min | 8.9 ± 3.1 (n = 62) | 8.8 ± 3.0 (n = 65) | −0.1 (−1.2 to 0.9) | 0.781 |
| Intubation | 8.8 ± 3.1 (n = 62) | 8.0 ± 2.6 (n = 65) | −0.8 (−1.8 to 0.2) | 0.105 |
| Intubation +1 min | 7.6 ± 3.0 (n = 62) | 7.0 ± 3.1 (n = 65) | −0.6 (−1.7 to 0.5) | 0.287 |
| Intubation +2 min | 8.3 ± 3.9 (n = 62) | 7.4 ± 3.1 (n = 65) | −0.8 (−2.0 to 0.4) | 0.194 |
| Intubation +3 min | 8.3 ± 3.2 (n = 62) | 7.6 ± 2.9 (n = 65) | −0.7 (−1.8 to 0.4) | 0.196 |
| Intubation +4 min | 8.7 ± 2.9 (n = 62) | 8.2 ± 3.0 (n = 65) | −0.5 (−1.5 to 0.6) | 0.353 |
| Intubation +5 min | 8.5 ± 2.7 (n = 62) | 8.0 ± 2.4 (n = 65) | −0.6 (−1.5 to 0.3) | 0.222 |
| Skin incision | 7.8 ± 2.4 (n = 62) | 7.8 ± 2.3 (n = 65) | 0.0 (−0.8 to 0.8) | 0.955 |
| Wound closing | 6.8 ± 1.9 (n = 62) | 7.1 ± 1.6 (n = 65) | 0.3 (−0.3 to 0.9) | 0.329 |
| Skin suturing | 6.4 ± 1.8 (n = 62) | 6.9 ± 1.7 (n = 65) | 0.5 (−0.1 to 1.1) | 0.103 |
| End of surgery | 6.3 ± 2.0 (n = 62) | 6.6 ± 1.7 (n = 65) | 0.3 (−0.3 to 1.0) | 0.298 |
| Extubation | 5.0 ± 1.7 (n = 62) | 5.6 ± 2.0 (n = 65) | 0.6 (0.0 to 1.3) | 0.063 |
| Recorded exit | 5.5 ± 1.6 (n = 62) | 5.7 ± 1.7 (n = 65) | 0.3 (−0.3 to 0.8) | 0.383 |

Group summaries are mean ± SD with observed n. Baseline values are descriptive; no baseline contrasts or P values are presented. Post-baseline mean differences and 95% CIs use Welch calculations; P values are unadjusted pointwise descriptions. Landmarks are categorical, not equally spaced elapsed-time observations. The turning landmark is omitted because its meaning differs or is unverified across patients. “Recorded exit” does not imply verified PACU discharge.

# Table S2. Repeated-measures models and estimands

| **Outcome / model** | **LR χ²** | **df** | **P** | **AIC** | **BIC** |
| --- | --- | --- | --- | --- | --- |
| MAP / Random intercept / CS | 10.10 | 12 | 0.607 | 13480.4 | 13560.1 |
| MAP / Unstructured covariance | 8.81 | 12 | 0.719 | 13023.1 | 13355.9 |
| HR / Random intercept / CS | 11.76 | 12 | 0.465 | 11924.0 | 12003.6 |
| HR / Unstructured covariance | 16.29 | 12 | 0.178 | 11045.5 | 11378.3 |
| BIS / Random intercept / CS | 120.28 | 12 | <0.001 | 12167.8 | 12247.4 |
| BIS / Unstructured covariance | 55.00 | 12 | <0.001 | 10866.2 | 11198.9 |

The common unstructured covariance has 91 free covariance parameters; its full group × landmark model has 26 mean parameters. The random-intercept model uses a compound-symmetry (CS) covariance. Both full and reduced models use ML, with 12 interaction degrees of freedom. AIC and BIC are computed from the same multivariate Gaussian likelihood; BIC uses 127 independent participants. These model comparisons use all 13 complete induction landmarks, including baseline. All reported fits converged; unstructured covariance condition numbers were 72.0 (MAP), 218.3 (heart rate), and 76.7 (BIS).

| **cLDA average post-baseline contrast** | **Estimate** | **95% CI** | **P** |
| --- | --- | --- | --- |
| MAP | 1.71 | −2.04 to 5.46 | 0.371 |
| HR | −0.65 | −3.29 to 2.00 | 0.632 |
| BIS | −5.86 | −7.82 to −3.89 | <0.001 |

cLDA constrains the population baseline mean to be common across groups and estimates 12 post-baseline group contrasts using a common unstructured covariance. The reported estimand is their equally weighted average. Wald CIs are model-based; no Kenward–Roger small-sample correction is claimed. The MAP estimate is 1.71 mmHg (95% CI, −2.04 to 5.46). For comparison, the robust unadjusted average over the 12 post-baseline landmarks is 2.62 mmHg (95% CI, −1.34 to 6.58), and the unadjusted average over all 13 landmarks is 2.76 mmHg (95% CI, −1.09 to 6.61; P = 0.160). The latter is not a baseline-adjusted treatment effect.

| **GEE model** | **Patients / readings** | **Wald χ²** | **df** | **P** |
| --- | --- | --- | --- | --- |
| MAP / 19 common landmarks | 127/2412 | 26.37 | 18 | 0.092 |
| MAP / Age 40–80, 13 landmarks | 120/1560 | 10.77 | 12 | 0.549 |
| HR / 19 common landmarks | 127/2413 | 30.84 | 18 | 0.030 |
| HR / Age 40–80, 13 landmarks | 120/1560 | 19.20 | 12 | 0.084 |
| BIS / 19 common landmarks | 127/2413 | 85.95 | 18 | <0.001 |
| BIS / Age 40–80, 13 landmarks | 120/1560 | 63.93 | 12 | <0.001 |

Gaussian GEE uses independent working correlation with participant-cluster robust covariance. The sandwich estimator allows within-patient correlation without imposing equal elapsed-time spacing. Missing observations are omitted; this approach does not eliminate bias from informative missingness. Full-course and age-eligible analyses were added during revision.

# Table S3. Documented induction vasoactive treatment

| **Agent** | **P: patients with a note** | **PS: patients with a note** |
| --- | --- | --- |
| Norepinephrine | 2 | 5 |
| Methoxamine | 9 | 6 |
| Dopamine | 11 | 7 |
| Epinephrine | 0 | 1 |
| Atropine | 0 | 1 |

At least one vasoactive agent other than atropine was documented in 17 P and 12 PS patients during induction. Agent counts overlap. These are minimum documented patient counts and must not be interpreted as complete treatment incidences; no between-group hypothesis tests are applied. Notes are anchored to recorded landmarks, not necessarily to the exact administration time. Rates in mL/h cannot be converted to mass without concentrations, and duration is incomplete. Atropine is presented separately as chronotropic treatment.

## Documented patient–agent entries

| **Study ID** | **Agent** | **Recorded landmark(s)** | **Recorded dose/rate text** |
| --- | --- | --- | --- |
| P-004 | Norepinephrine | Intubation +2 min | Norepinephrine 6ml/h infusion |
| P-030 | Norepinephrine | Intubation +5 min | Norepinephrine 5ml/h infusion |
| PS-007 | Norepinephrine | Intubation +5 min | Norepinephrine 5ml/h infusion |
| PS-008 | Norepinephrine | Intubation +2 min | Norepinephrine 5ml/h infusion |
| PS-010 | Norepinephrine | Drug +2 min | Norepinephrine 20ml/h infusion |
| PS-030 | Norepinephrine | Drug +4 min; Drug +5 min | Norepinephrine 2ug; Norepinephrine 5ml/h infusion |
| PS-034 | Norepinephrine | Intubation +3 min | Norepinephrine infusion5ml/h |
| P-021 | Methoxamine | Drug +5 min | Methoxamine 15ml/h infusion |
| P-022 | Methoxamine | Intubation +2 min | Methoxamine 10ml/h infusion |
| P-025 | Methoxamine | Drug +4 min | Methoxamine 20ml/h infusion |
| P-035 | Methoxamine | Intubation +5 min | Methoxamine 20ml/h infusion |
| P-039 | Methoxamine | Intubation +2 min | Methoxamine 20ml/h infusion |
| P-043 | Methoxamine | Drug +3 min | Methoxamine 20ml/h infusion-13:55 |
| P-044 | Methoxamine | Drug +2 min | Methoxamine 20ml/h infusion-10:30 |
| P-053 | Methoxamine | Drug +5 min | Methoxamine 20ml/h-9:00 |
| P-062 | Methoxamine | Drug +5 min | Methoxamine 30ml/h-10:00 |
| PS-005 | Methoxamine | Drug +5 min; Intubation +3 min | Methoxamine infusion; stop Methoxamine |
| PS-010 | Methoxamine | Drug +3 min | Methoxamine 30ml/h infusion |
| PS-037 | Methoxamine | Intubation +3 min | Methoxamine 10ml/h infusion |
| PS-043 | Methoxamine | Drug +4 min | Methoxamine 20ml/h infusion |
| PS-058 | Methoxamine | Intubation +5 min | Methoxamine 20ml/h infusion-9:40 |
| PS-059 | Methoxamine | Drug +3 min | Methoxamine 20ml/h infusion-12:00 |
| P-002 | Dopamine | Intubation +3 min | Dopamine 2mg IV bolus |
| P-003 | Dopamine | Drug +4 min; Intubation +2 min | Dopamine 2mg IV bolus |
| P-004 | Dopamine | Drug +1 min | Dopamine 2mg IV bolus |
| P-022 | Dopamine | Intubation +1 min | Dopamine 2mg IV bolus |
| P-025 | Dopamine | Drug +3 min | Dopamine 2mg IV bolus |
| P-039 | Dopamine | Drug +4 min | Dopamine 2mg |
| P-046 | Dopamine | Intubation +4 min | Dopamine 2mg |
| P-048 | Dopamine | Intubation +3 min | Dopamine 2mg |
| P-052 | Dopamine | Intubation +2 min | Dopamine 2mg |
| P-061 | Dopamine | Intubation +4 min | Dopamine 2mg |
| P-062 | Dopamine | Drug +3 min; Intubation +4 min | Dopamine 2mg |
| PS-007 | Dopamine | Drug +3 min | Dopamine 2mg IV bolus |
| PS-008 | Dopamine | Drug +3 min; Intubation +1 min | Dopamine 2mg IV bolus |
| PS-030 | Dopamine | Drug +3 min | Dopamine 2mg |
| PS-039 | Dopamine | Drug +4 min | Dopamine 2mg IV bolus |
| PS-042 | Dopamine | Drug +4 min | Dopamine 2mg IV bolus |
| PS-058 | Dopamine | Intubation +4 min | Dopamine 2mg |
| PS-059 | Dopamine | Drug +2 min; Intubation +3 min | Dopamine 2mg |
| PS-010 | Epinephrine | Drug +4 min; Intubation; Intubation +2 min | Epinephrine 20ug IV bolus; Epinephrine 10ug IV bolus |
| PS-005 | Atropine | Drug +4 min | Atropine 0.5mg |

Study IDs are audit pseudonyms. “Stop” entries denote a documented stop, not a new dose. This listing preserves ambiguous dose/rate strings rather than assigning units or concentrations that were not recorded. It is an audit of retained notes, not a reconstructed medication administration record.

# Table S4. Fluid summaries and available denominators

| **Outcome** | **Group P** | **Group PS** | **MD (95% CI)** | **P value** |
| --- | --- | --- | --- | --- |
| Recorded crystalloid (mL) | 1131.5 ± 353.2 (n = 62) | 1213.3 ± 398.7 (n = 64) | 81.8 (−50.9 to 214.6) | 0.225 |
| Recorded total input (mL) | 1253.7 ± 390.1 (n = 62) | 1342.1 ± 577.5 (n = 65) | 88.4 (−84.2 to 260.9) | 0.312 |
| Recorded blood loss (mL) | 116.0 ± 169.2 (n = 62) | 120.3 ± 197.3 (n = 65) | 4.3 (−60.1 to 68.8) | 0.894 |
| Recorded urine output (mL) | 340.3 ± 314.6 (n = 62) | 306.9 ± 347.2 (n = 65) | −33.4 (−149.7 to 82.9) | 0.571 |
| Recorded total output (mL) | 456.8 ± 393.1 (n = 62) | 427.2 ± 465.5 (n = 65) | −29.5 (−180.6 to 121.6) | 0.699 |
| Recorded net balance (mL) | 796.9 ± 306.4 (n = 62) | 914.8 ± 336.6 (n = 65) | 117.9 (5.0 to 230.9) | 0.041 |

Values are mean ± SD with observed n; MD and CIs use Welch calculations. Net balance is recorded total input minus recorded total output. One PS crystalloid cell is missing. Component amounts are not inferred from totals. Colloid amounts were populated for only 18 P and 14 PS patients (observed means 400.0 and 417.0 mL); absent component entries were not treated as verified zeros or as documented non-exposure. Blood-product component blanks were likewise not assumed to be zero. The timing of fluid administration is unavailable, so the net-balance contrast cannot establish its contribution to MAP.

# Table S5. Source-data issues and sensitivity analyses

| **Issue** | **Source observation** | **Analytical handling** |
| --- | --- | --- |
| Induction propofol | P-030, AF10 empty | n = 61 for P induction; no imputation from total minus maintenance. |
| Phase/total inconsistency | P-060: 120 + 250 mg; total 320 mg | Retain recorded total in main analysis; exclude record and arithmetic 370-mg alternatives only in sensitivity analyses. |
| Internal BP inconsistency | P-023, Y79 MAP 6; Y78 BP 105/51 | Retained and flagged. Neither MAP nor paired BP is treated as verified truth. |
| MAP range checks | 29 readings / 25 patients outside paired range; 20 / 19 with >5-mmHg tolerance | Mask only flagged MAP in two exploratory GEE sensitivities; retain all patients. |
| Induction timestamps | Strictly increasing in 54/62 P and 63/65 PS | Eight P and one PS have duplicate-minute intervals; one PS has a reversal. Exclude only from the clock-based area; rounding may explain duplicates. |
| Other timestamps | 30 stated surgery durations differ from incision-to-end interval by >1 min | Retain recorded duration as labeled; do not silently substitute a different timing definition. PS-039 exit precedes extubation by 7 min; PS-049 incision precedes the preceding landmark by 166 min. |
| Turning landmark | Populated for 49 P and 52 PS, including two anterior-cervical cases per group | Exclude from 19 common-landmark analysis; no alternative landmark assigned. |
| Potassium | Baseline n = 61/65; extubation n = 58/62; paired n = 57/62 | No filling from exit or other landmarks. PS-006 has recorded potassium supplementation. |
| Early potassium entry | PS-002, D94 = 30 mmol/L | Uncorroborated implausible value; no substitution with 3.0. No usable systematic early series. |
| Anthropometry | P-028, height 115 cm; weight 85 kg | Retained and flagged. No source correction or alternative dosing weight was applied. |
| Age eligibility | Five P and two PS outside 40–80 years | Retain all 127 in main analysis; age-eligible sensitivity uses 120. |
| Collection and coding | Two retained Excel workbooks; blanks and numeric strings | Numeric strings with explicit totals retain the value after “=” (e.g., 80+150=230); comma-decimal 4,14 and 4,73 are parsed as 4.14 and 4.73. No source cells overwritten. |

# Table S5 (continued). Quantitative sensitivities

| **Outcome** | **Group P** | **Group PS** | **MD (95% CI)** | **P value** |
| --- | --- | --- | --- | --- |
| Total propofol: recorded source | 751.3 ± 412.2 (n = 62) | 515.4 ± 319.3 (n = 65) | −235.9 (−366.0 to −105.9) | <0.001 |
| Total propofol: omit inconsistent P record | 758.4 ± 411.9 (n = 61) | 515.4 ± 319.3 (n = 65) | −243.0 (−373.7 to −112.3) | <0.001 |
| Total propofol: arithmetic 370-mg alternative | 752.1 ± 411.4 (n = 62) | 515.4 ± 319.3 (n = 65) | −236.7 (−366.6 to −106.9) | <0.001 |
| Nadir MAP: source | 69.8 ± 14.0 (n = 62) | 72.8 ± 14.1 (n = 65) | 3.0 (−1.9 to 8.0) | 0.226 |
| Nadir MAP: strict range mask | 72.1 ± 10.3 (n = 62) | 74.2 ± 12.7 (n = 65) | 2.1 (−2.0 to 6.1) | 0.314 |
| Nadir MAP: >5-mmHg range mask | 71.7 ± 10.4 (n = 62) | 73.8 ± 13.3 (n = 65) | 2.1 (−2.1 to 6.3) | 0.325 |

The alternative 370-mg total is an arithmetic reconstruction, not a verified correction. It reproduces the former P mean of 752.1 mg, but does not establish how that earlier value was produced or which source entry was wrong. The main analysis therefore uses 751.3 mg. Replacing or excluding this one record does not remove the observed propofol difference.

| **Outcome** | **Group P** | **Group PS** | **MD (95% CI)** | **P value** |
| --- | --- | --- | --- | --- |
| Ordinal unit-spacing area (not mmHg·min) | 0.00 [0.00–3.00] (n = 62) | 0.00 [0.00–0.00] (n = 65) | 0.06 (−4.74 to 4.86) | 0.386 |
| Clock-based trapezoid of positive deficits | 0.00 [0.00–4.75] (n = 54) | 0.00 [0.00–0.25] (n = 63) | −1.06 (−8.00 to 5.88) | 0.298 |
| Clock-based piecewise-linear MAP area | 0.00 [0.00–2.22] (n = 54) | 0.00 [0.00–0.05] (n = 63) | 0.00 (−4.73 to 4.72) | 0.320 |

Area rows show median [IQR] and n; MD/CI describe mean differences, while P values use Mann–Whitney distributional comparisons. The first row is retained only to reproduce the former result and has no valid minute unit. The second joins positive deficit values directly and differs from integrating a linearly interpolated MAP when the threshold is crossed. The third is the exploratory result used in the revision. Its group means ± SD are 3.87 ± 9.64 and 3.86 ± 15.79 mmHg·min; zeros occur in 36/54 and 47/63 patients. Sparse landmark data and recording inconsistencies remain limitations under either interpolation rule.

MAP interaction sensitivity: Gaussian GEE P = 0.699 using all induction readings; P = 0.830 after strict MAP-range masking; P = 0.729 with the >5-mmHg rule. These analyses assess robustness to specified data-handling assumptions; they cannot verify the true BP readings or eliminate informative missingness.

# Interpretation of recorded extubation intervals

The surgery-end-to-extubation interval was derived during revision. Welch analysis estimates the difference in means (3.1 min; 95% CI, 0.9–5.2), whereas the Mann–Whitney test compares distributions (P = 0.074). Identical medians do not imply identical means or recovery profiles. The interval includes clinical decisions about extubation and does not directly measure return of consciousness. PS-039 had an exit timestamp preceding extubation by 7 min; this record was retained and is identified in Figure 6A. No verified replacement time was available.
